# Supplementary figures and images for: Isoflurane anesthesia alters 31P magnetic resonance spectroscopy markers compared to awake mouse brain
Source: PLoS One. 2025 Nov 14;20(11):e0333627. doi: 10.1371/journal.pone.0333627 (PMC12617894; doi:10.1371/journal.pone.0333627)

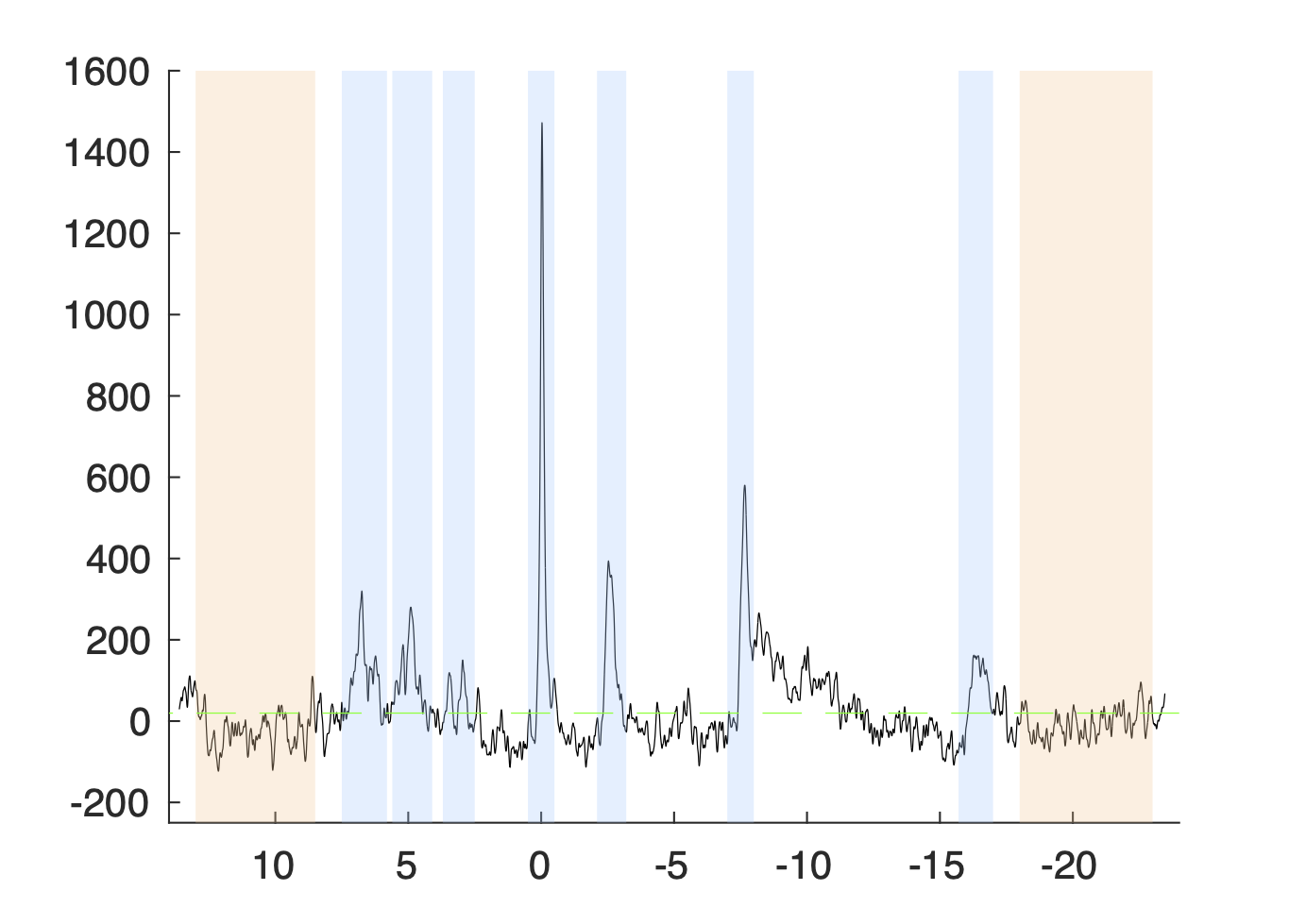

Supplement: S1 Fig — This figure illustrates the set ranges for each individual metabolite and the threshold (green dashed line) used calculate the AUC for each metabolite. (TIFF) [file pone.0333627.s001.tif]

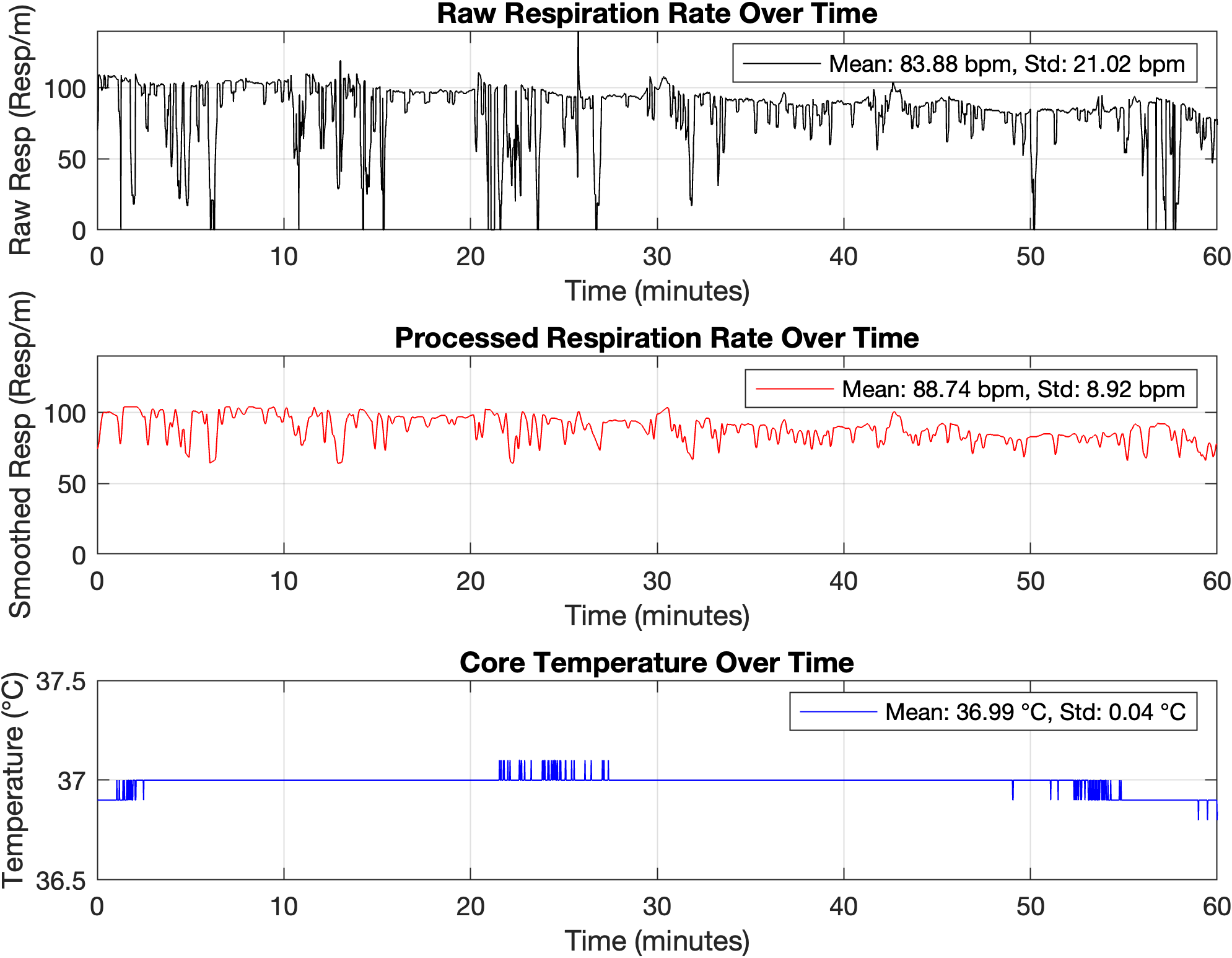

Supplement: S2 Fig — Temperature measurements are unprocessed and include only measurements during ISIS acquisition, while respiratory data are processed. (TIFF) [file pone.0333627.s002.tif]

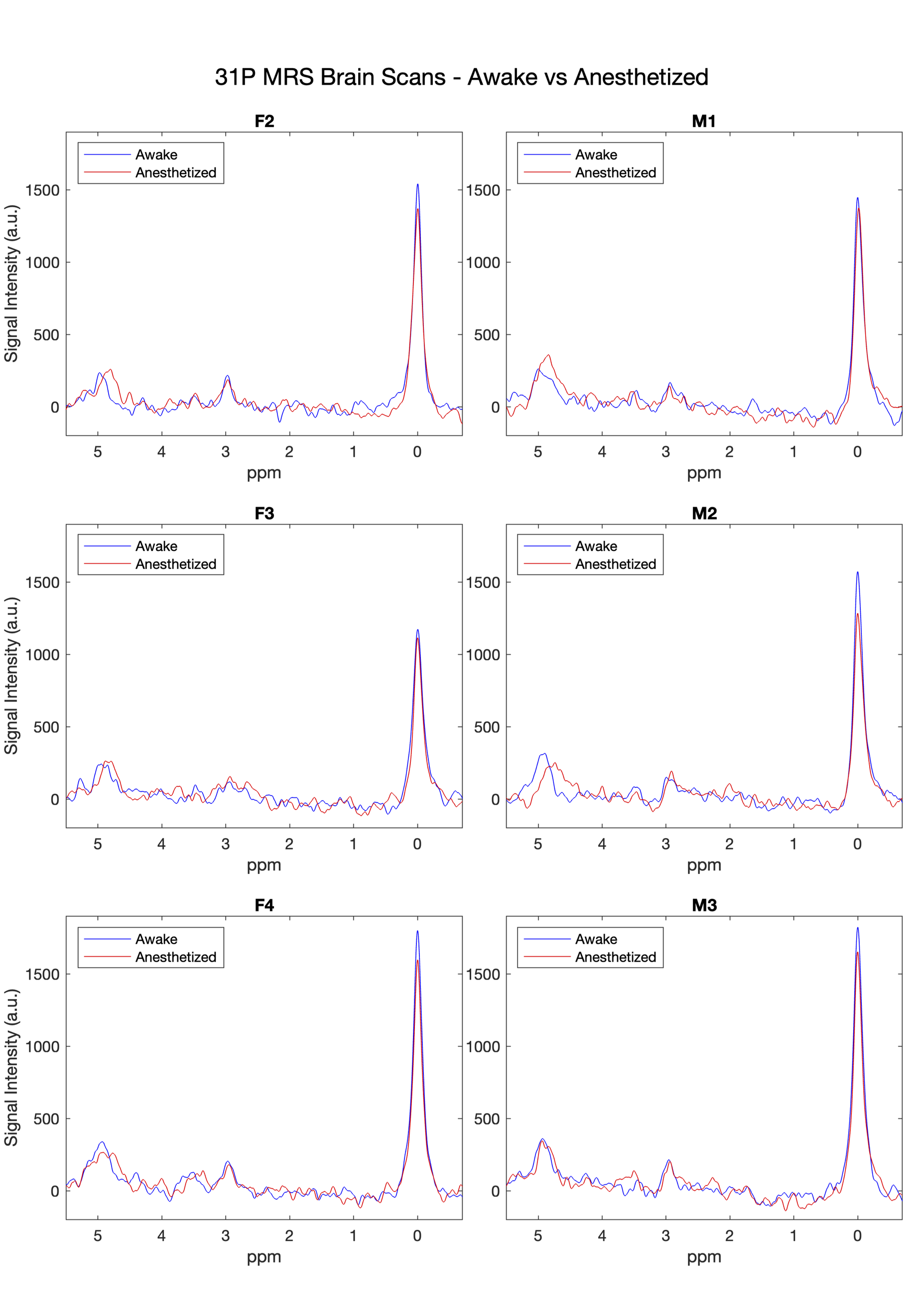

Supplement: S3 Fig — (TIFF) [file pone.0333627.s003.tif]

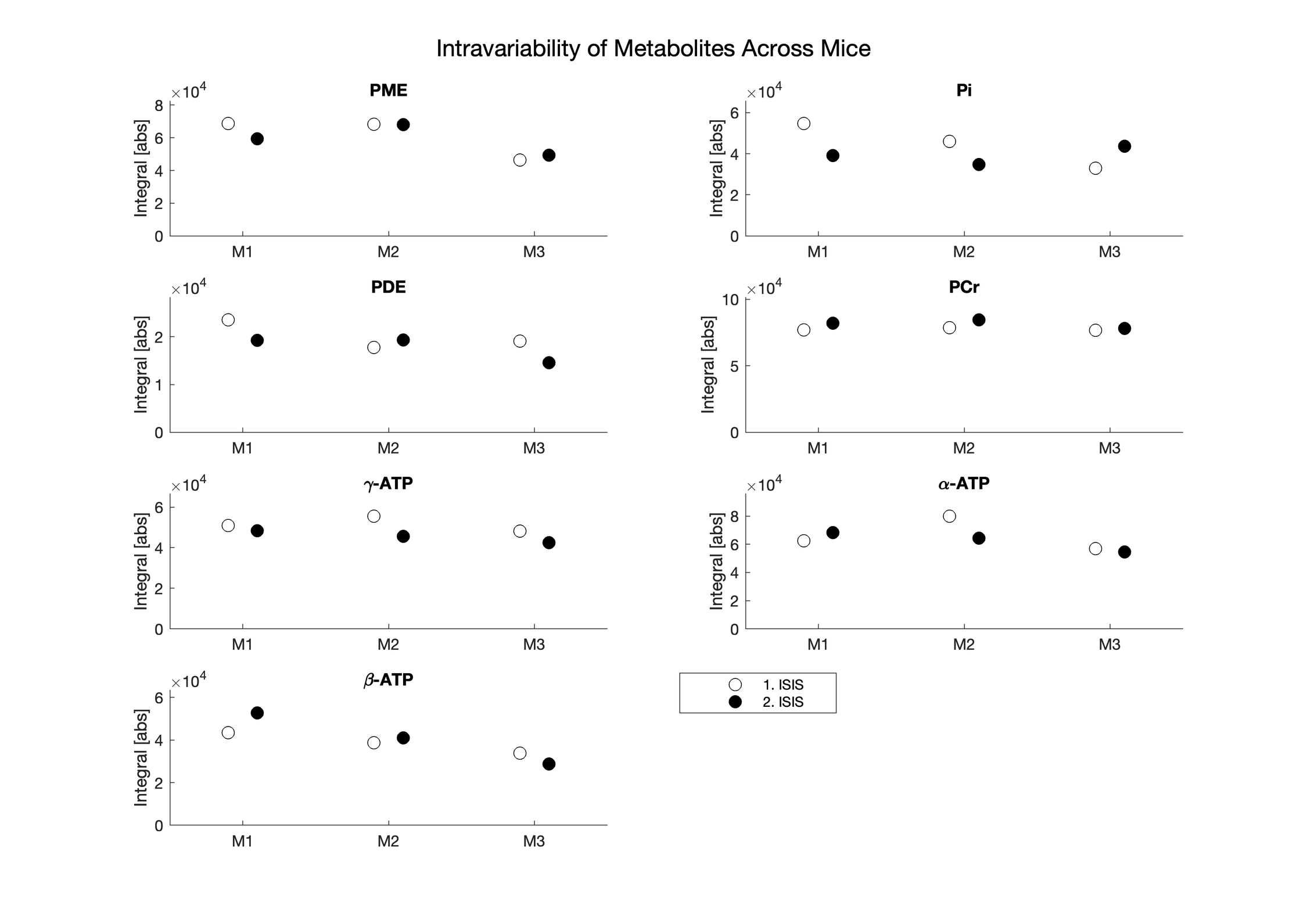

Supplement: S4 Fig — (TIFF) [file pone.0333627.s004.tif]

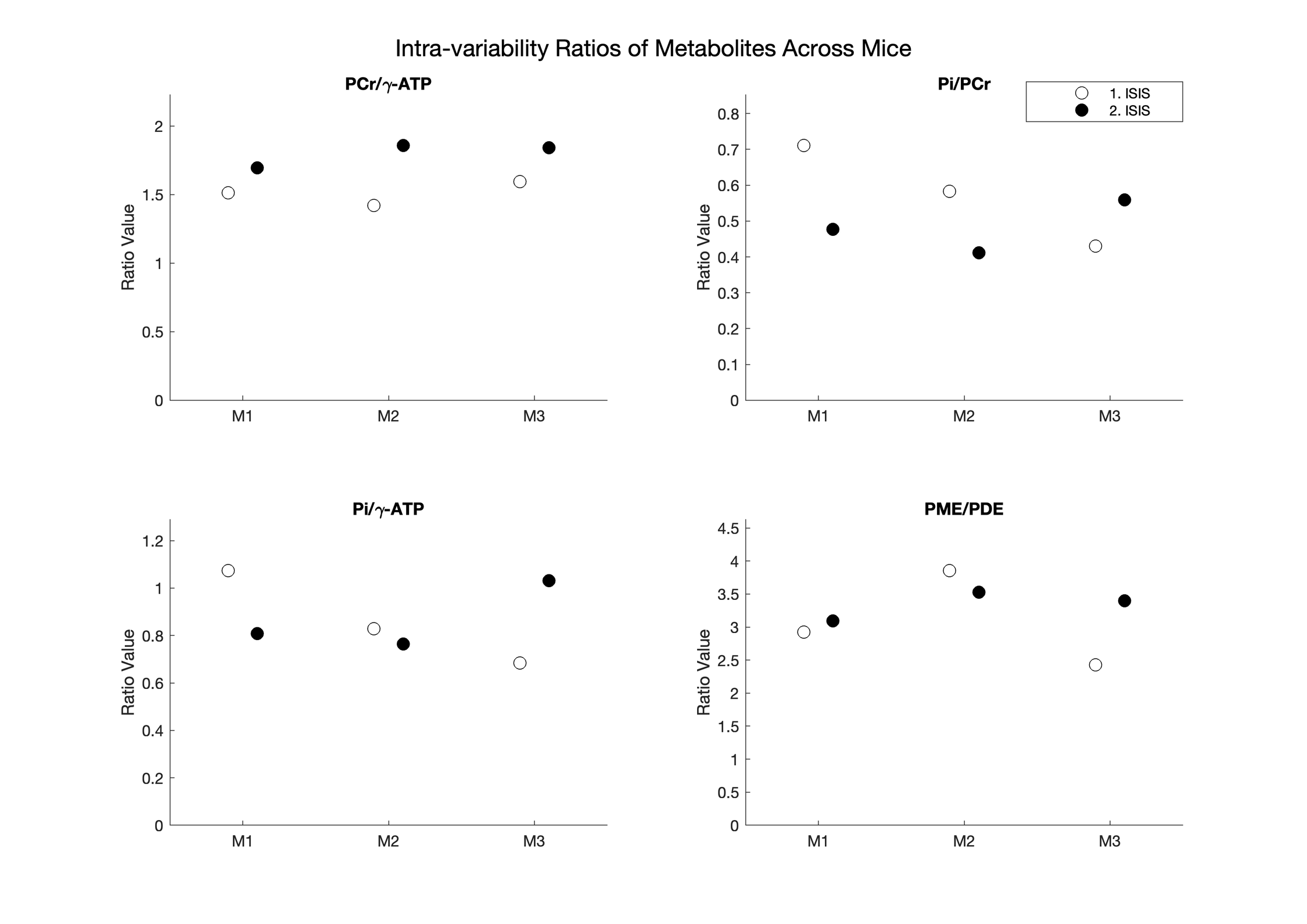

Supplement: S5 Fig — (TIFF) [file pone.0333627.s005.tif]

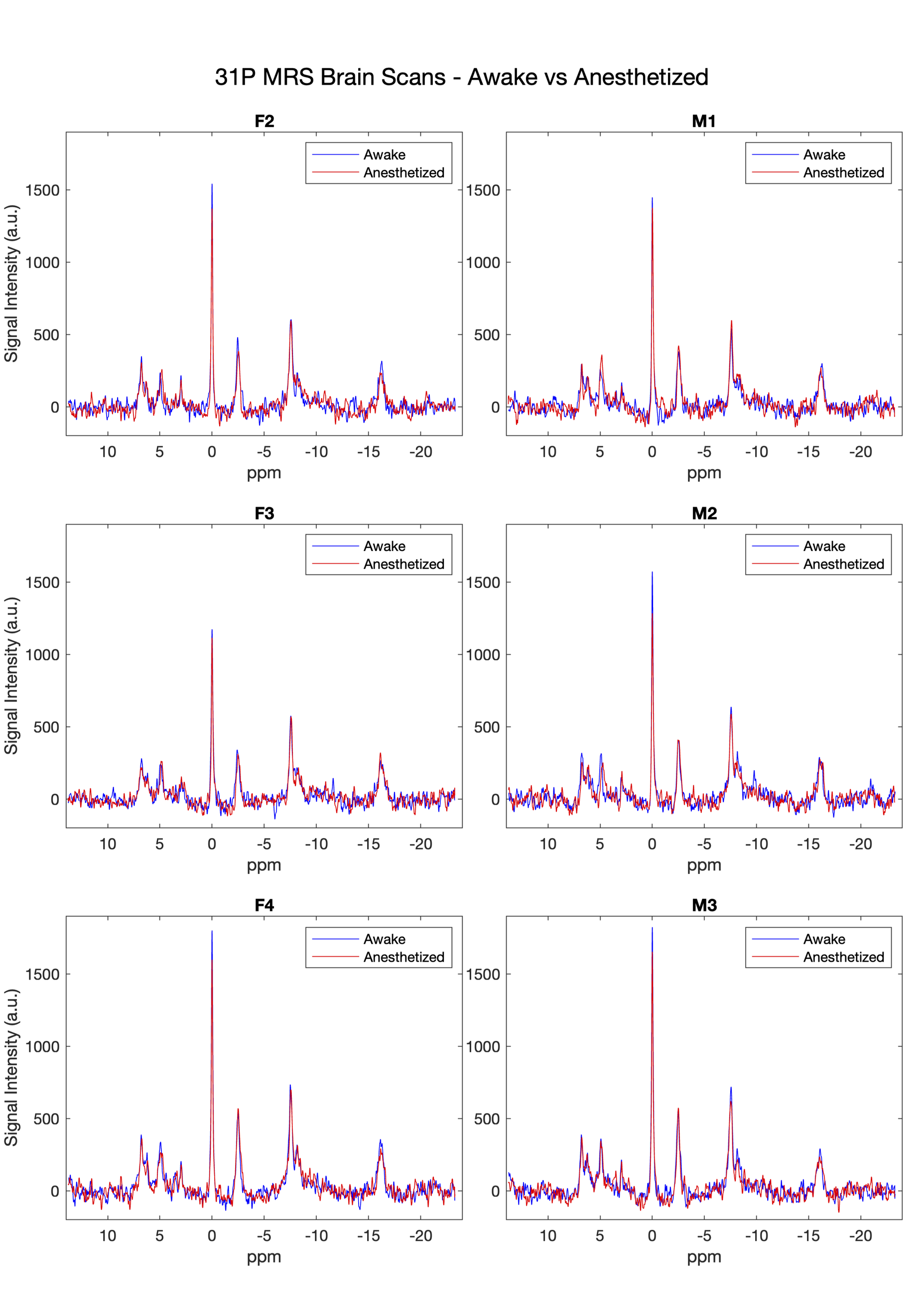

Supplement: S6 Fig — Each individual 31P Spectrum from male and female mice in both awake and anesthetized states. (TIFF) [file pone.0333627.s006.tif]

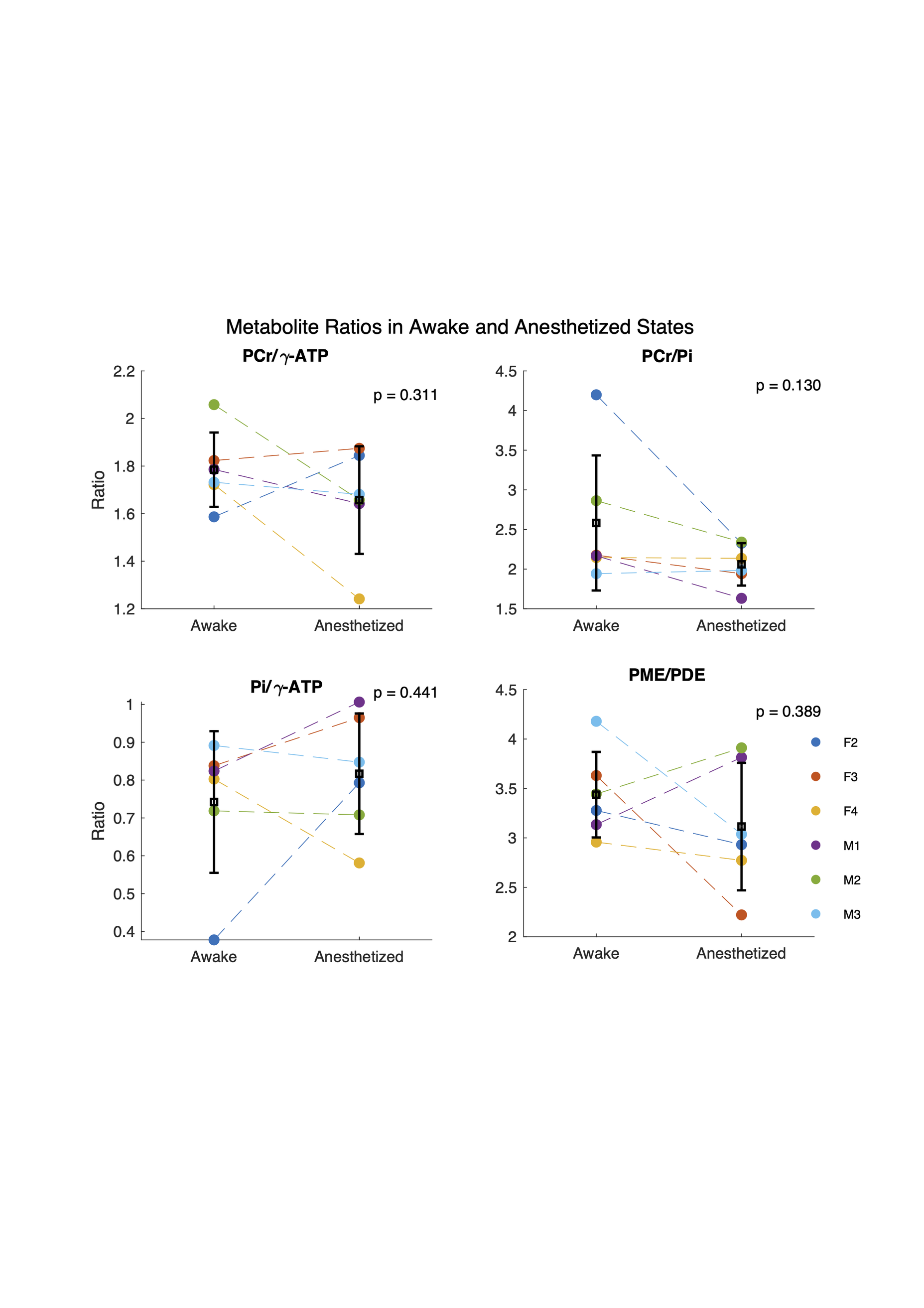

Supplement: S7 Fig — The AUC where used to determine the PCr/γ-ATP and PCr/Pi ratios. No significant changes where found when comparing awake and anesthetized states. (TIFF) [file pone.0333627.s007.tif]

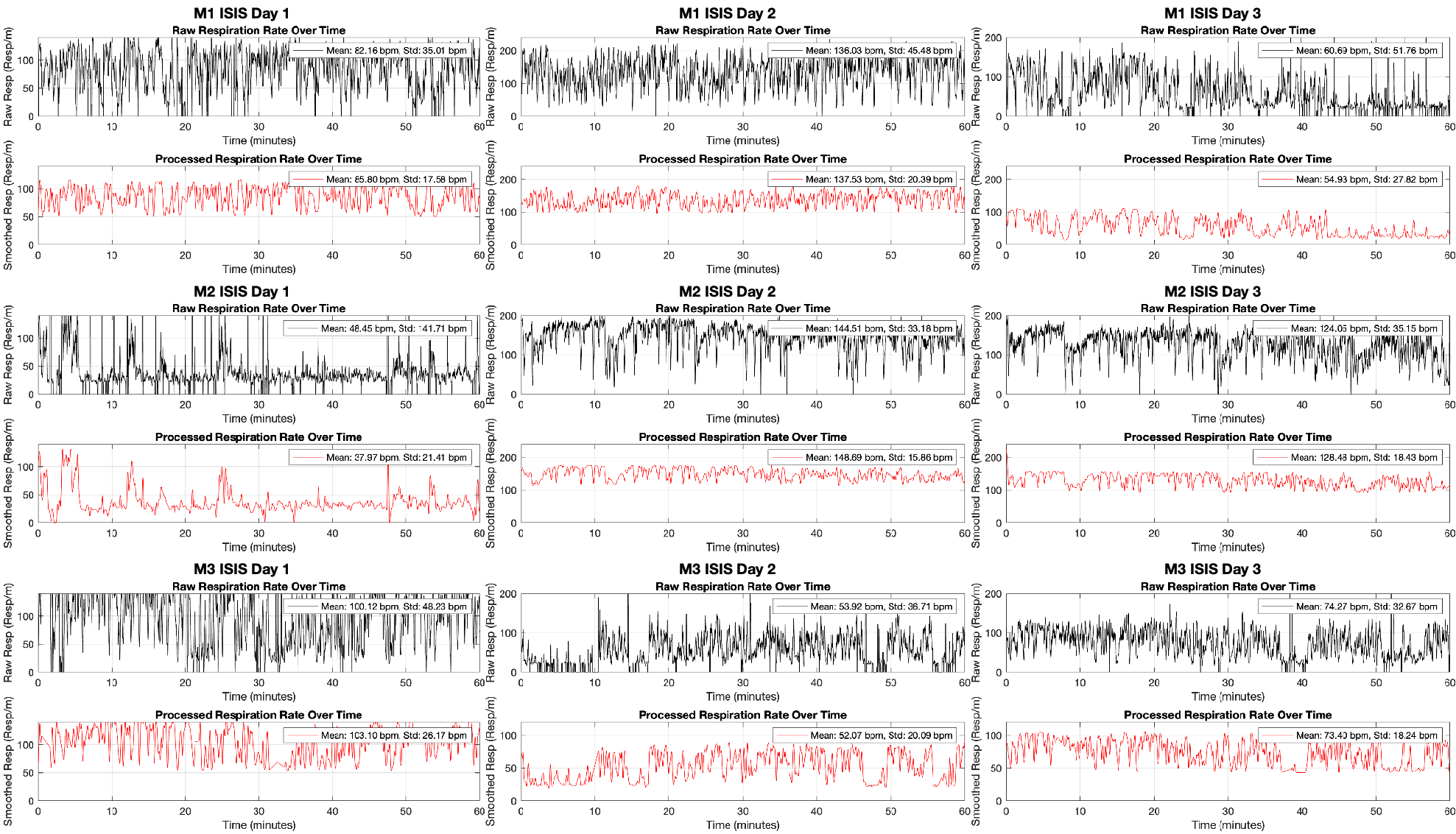

Supplement: S8 Fig — (TIFF) [file pone.0333627.s008.tif]
