# Supplementary material for: Isoflurane anesthesia alters 31P magnetic resonance spectroscopy markers compared to awake mouse brain
Source: PLoS One. 2025 Nov 14;20(11):e0333627. doi: 10.1371/journal.pone.0333627 (PMC12617894; doi:10.1371/journal.pone.0333627)
Supplement: Attachment — Submitted filename: PLOSONE_Review_31PawakeMouse.docx [file pone.0333627.s015.docx]

The manuscript “Isoflurane anesthesia alters 31P Magnetic Resonance Spectroscopy markers compared to awake mouse brain” presents a technically sound study comparing brain energy metabolism in awake versus isoflurane-anesthetized mice using 31P-MRS. The authors demonstrate that even low-dose isoflurane significantly reduces phosphocreatine (PCr) levels and intracellular pH, without affecting ATP concentrations or PCr/ATP ratios. The study is strengthened by meticulous attention to experimental detail, e.g. in the design of the custom head-fixation and cradle system, animal habituation protocol, 31P power calibration and, in particular, the careful control of physiological parameters. These efforts convincingly establish the feasibility of awake 31P-MRS and provide a reference point for future studies on brain metabolism under physiological versus anesthetized conditions. While the methodology is robust, several aspects — such as repeatability under awake conditions and the interpretation of specific metabolic shifts and the extensive biological interpretations in the discussion would strongly benefit from a larger number of animals and/or experiments.

However, some minor issues and questions remain:

Many of the Figure and suppl. Figure references as well as the references to suppl. Stl files are wrong. I have marked the ones I saw, but please revise the manuscript carefully.

Animal prep, Page 4, line 140:

Please clarify the timing of the carprofen, Buprenorphine and ampicillin. In the text it read like it was injected i.p. right before incision. However at least for Buprenorphine and ampicillin recommendations are 30-60min prior to surgery. Also most of the drugs are recommended s.c. Why did you decide for i.p.?

Caption Fig 1: “… supplementary files 4 4.” The Manuscript has stl files called, e.g., ‘MR head plate.stl’

please fix reference.

Page 5, Caption Fig 2: again the reference to supplementary files “4, 4.” please fix.

Page 6, Caption Fig 3: again the reference to supplementary files “4.” please fix.

Line 219: see Fig 4 should be Fig 2?

Page 7, line 239ff: with an ISIS scan time of 60min, why was it impossible to do two awake acquisitions back to back? The duration of “awake MRI” probably starts the moment the mice are put into the head mount. However, thanks to the careful prior calibration of the 1H and 31P Reference power, the setup time (localizers) shouldn’t be that long?

Page 8, line 287: ref to Suppl. Fig 4 is wrong,. supplied SFig 4 is biometric results. Please fix.

line 297ff: It seems the respiration pad was not only used for online monitoring but was recorded. By	which system? Please add in methods.

line 300ff: Was this respiration processing performed on both awake and anesthetized? Which respiration

rates (awake or anesthetized) are shown in Sfig 4 (see also Caption on page 14) ? Since its a

specific animal, which one (M1..3, F1..3) ?

Page 9, line 319: again a wrong reference to suppl. Fig 4. please fix.

Table 1: Please try to add a visual indication which values are compared for the significant t-test results for	better understanding, e.g. mark both compared values by the same marker (its only two pairs).

Line 320, 324: Fig X → Fig 5? Please fix

Line 333ff: These are the RR under anesthesia. What were the RR in awake state? Did those also change	over time (habituation, increasing discomfort/panic, …) ?

Page 10, Caption fig 9: wrong reference to Sfig 4, please fix.

Line 352: References to Fig 9a and 9b – but figure doesn’t have a and b label. Please fix.

Chapter discussion:

There are almost two pages of discussion of the results and their biological interpretation, containing elaborate arguments, thought chains and connections with other known facts, which is appreciated. But it also sometime seems a bit of overinterpreting the results of a lower PCr content in awake mice with an n=3, while ATP and ratios were non significant. I do appreciate tying the PCr change and the pH change together with other prior knowledge, but maybe there is potential to shorten this and tighten the arguments?

Line 368: please fix typo texttimes

Page 12, line 452, 460: Is RR in line 452 a general value? Or measured in your experiment?

please add the measured RR values from awake state as comparison for the discussion about RRs	in anesthesia.

Page 13, line 473ff: the authors suggest awake mouse MT-31P-MRS should facilitate measuring the true, physiological ATP creation rates without isoflurane influence. This seems possible, but a few problems might be addressed as well, like limited scantime and MT-31P-MRS beeing notoriously long scans. Motion stability for longer scantimes in ISIS might still become a problem, even if its not actual head motion, but e.g. body motion during breathing or by fighting against the restraint, which could causie B0 fluctuations in the head. Please address these issues as well.

Line 479: Here authors claim, ATP suppression by anesthesia is substantial – however, that is not based on shown data nor is it backed by a reference. Please add support for that claim.

Line 483 needs a closing paranthesis after the figure 7 reference.

Line 482ff: While the argument, that basic 31P MRS analysis was already able to show significant PCr decrease, a more fancy analysis might have been able to find more accurate ATP changes and PCr/ATP ratios as well. However, the small n=3 would still weaken those results, so maybe indeed leave it at this.

Section “Methodological considerations”

In fMRI in rats and mice isoflurane does indeed vasodilate the brain vessels and leaves no no capacity to further open up the vessels for a increased blood flow in BOLD experiments, effectively canceling BOLD signal for isoflurane at 2%. However, a combination anesthesia of Medetomidine together with (very) low levels of isoflurane recover the BOLD responsiveness of the rodent brain. In comparison to your (low) isoflurane protocol, added Medetomidine might allow to lower isoflurane even further, maybe keeping the respiration rate >100, possibly also reducing the ATP suppression. Please add a discussion of regarding this well described anesthesia combination.

Page 14: author contributions

Line 523: … surgeries, SM, SM and RWK performed ..... : the second SM is not required/wrong, please fix

Support information:

The naming of the S1 File, S2 file is not the same as on the downloadable files, please fix

References:

Some references contain broken html formatting codes (e.g. 21 !sup?14 with ! And ? Inverted), please fix.

Also Ref 29, 71, 72, 88
