# Supplementary material for: Isoflurane anesthesia alters 31P magnetic resonance spectroscopy markers compared to awake mouse brain
Source: PLoS One. 2025 Nov 14;20(11):e0333627. doi: 10.1371/journal.pone.0333627 (PMC12617894; doi:10.1371/journal.pone.0333627)
Supplement: Attachment — Submitted filename: Response to reviewers.docx [file pone.0333627.s016.docx]

**We thank the reviewers for their thorough and insightful review of our manuscript. Their input has helped improve the study and we hope the reviewers agree that the revised version is much improved. Point-by-point responses are provided below and changes are clearly marked in the revised manuscript.**

**It is our hope that the manuscript is now deemed ready for publication in PLoS1.**

**Reviewer 1**

The manuscript “Isoflurane anesthesia alters 31P Magnetic Resonance Spectroscopy markers compared to awake mouse brain” presents a technically sound study comparing brain energy metabolism in awake versus isoflurane-anesthetized mice using 31P-MRS. The authors demonstrate that even low-dose isoflurane significantly reduces phosphocreatine (PCr) levels and intracellular pH, without affecting ATP concentrations or PCr/ATP ratios. The study is strengthened by meticulous attention to experimental detail, e.g. in the design of the custom head-fixation and cradle system, animal habituation protocol, 31P power calibration and, in particular, the careful control of physiological parameters. These efforts convincingly establish the feasibility of awake 31P-MRS and provide a reference point for future studies on brain metabolism under physiological versus anesthetized conditions. While the methodology is robust, several aspects — such as repeatability under awake conditions and the interpretation of specific metabolic shifts and the extensive biological interpretations in the discussion would strongly benefit from a larger number of animals and/or experiments.

However, some minor issues and questions remain:

Many of the Figure and suppl. Figure references as well as the references to suppl. Stl files are wrong. I have marked the ones I saw, but please revise the manuscript carefully.

**Agreed, this has been corrected throughout.**

Animal prep, Page 4, line 140:

Please clarify the timing of the carprofen, Buprenorphine and ampicillin. In the text it read like it was injected i.p. right before incision. However at least for Buprenorphine and ampicillin recommendations are 30-60min prior to surgery. Also most of the drugs are recommended s.c. Why did you decide for i.p.?

**Our protocol is made with the guidance by the veterinarians, and their recommendation was to use ip. injection on the day of surgery because this allows the drugs take effect sooner. In all cases, there was at least 30 min from injection to the beginning of the first incision. Post Op medication was administrered sc. These details have been added for clarity.**

Caption Fig 1: “… supplementary files 4 4.” The Manuscript has stl files called, e.g., ‘MR head plate.stl’ please fix reference.

**Done.**

Page 5, Caption Fig 2: again the reference to supplementary files “4, 4.” please fix.

**Done.**

Page 6, Caption Fig 3: again the reference to supplementary files “4.” please fix.

Line 219: see Fig 4 should be Fig 2?

**The reference to Supplementary file has been corrected. Figure 4 shows the placement of the voxel, which is why we refer to it when explaining the avoidance of non-brain tissue in line 219.**

Page 7, line 239ff: with an ISIS scan time of 60min, why was it impossible to do two awake acquisitions back to back? The duration of “awake MRI” probably starts the moment the mice are put into the head mount. However, thanks to the careful prior calibration of the 1H and 31P Reference power, the setup time (localizers) shouldn’t be that long?

**That is a very good observation; however, there are the following limitations. The B0 map involves three averages, and together with the required FOV and image resolution takes 15 minutes to acquire, and it must be performed twice (once prior to each ISIS acquisition) to account for drift of the magnetic field. This alone accounts for 2.5 hours, excluding the time needed for voxel placement, quality control of the B0 map, and a few minutes in total for some minimal manual steps for each scan sequence. In anesthetized mice, the total scan time ranged from 2 hours 45-55 minutes for back-to-back ISIS scans. Although anesthesia was administered only briefly to allow placement of the mouse in the cradle, we allowed the mice 10 minutes to recover before performing the B0 map, giving them a total of app. 30 minutes before starting the ISIS scan after the cessation of isoflurane. This means that, in total, we would need at least 3 hours to perform back-to-back awake ISIS scans. This exceeds the time allowed for awake experiments by the ethics committee.**

Page 8, line 287: ref to Suppl. Fig 4 is wrong,. supplied SFig 4 is biometric results. Please fix.
**Done.**

line 297ff: It seems the respiration pad was not only used for online monitoring but was recorded. By	which system? Please add in methods.

**Done.**

l

Line 300ff: Was this respiration processing performed on both awake and anesthetized? Which respiration rates (awake or anesthetized) are shown in Sfig 4 (see also Caption on page 14) ? Since its a specific animal, which one (M1..3, F1..3) ?

**The specific mouse and experiment have been added. Respiration was recorded during the anesthetized states. This is due to awake scans involve more movement, and respiration signal loss is therefore more frequent. A respiration pad, as we used, or the MouseOx system are suboptimal for recording in awake mice, with the MouseOx being particularly difficult to set up inside the MR scanner. It is highly labor-intensive and would have extended both the overall preparation and scan duration, why we chose the pad. Both systems rely on maintaining a stable contact to the surface area and do not adapt to the skin as the mouse makes very small movements. Other systems, such as ECG or EMG needle electrodes for rodents, circumvent this issue; however, they are invasive, as the needles puncture the skin, and are not compatible with awake. Having a commercially available device with non-invasive, MR-compatible ECG electrodes would allow for robust and reliable recordings of both respiration and heart rate in awake mice, but such a device is, to our knowledge, not currently available.**

Page 9, line 319: again a wrong reference to suppl. Fig 4. please fix.

**Done.**

Table 1: Please try to add a visual indication which values are compared for the significant t-test results for	better understanding, e.g. mark both compared values by the same marker (its only two pairs).

**Done.**

Line 320, 324: Fig X → Fig 5? Please fix
**Done.**

Line 333ff: These are the RR under anesthesia. What were the RR in awake state? Did those also change	over time (habituation, increasing discomfort/panic, …) ?

**As previously mentioned, RR during awake MR sessions are of poor quality due to the technical limitations of current systems, which are unreliable even in awake, restraint-trained mice. However, we did manage to reliably assess the RR from five sessions showing that awake RR is in agreement with previously reported RR values in unstressed, awake mice. This is seen in Supplmenetary Fig. S8. This result is expected, of course, because we use the habituation protocol described by Lindhardt et al. (2022), who demonstrated a decrease in heart rate, and corticosterone levels over the course of habituation. See also response to R2 on similar matters.**

Page 10, Caption fig 9: wrong reference to Sfig 4, please fix.

**Done**

Line 352: References to Fig 9a and 9b – but figure doesn’t have a and b label. Please fix.

**Done.**

Chapter discussion:

There are almost two pages of discussion of the results and their biological interpretation, containing elaborate arguments, thought chains and connections with other known facts, which is appreciated. But it also sometime seems a bit of overinterpreting the results of a lower PCr content in awake mice with an n=3, while ATP and ratios were non significant. I do appreciate tying the PCr change and the pH change together with other prior knowledge, but maybe there is potential to shorten this and tighten the arguments?

**We agree that the Discussion was too long and overly broad. This concern was also raised by the other reviewer. In the revised manuscript we have shortened the Discussion and narrowed its scope. We hope the reviewer agrees that this has improved the manuscript.**

**With regard to the interpretation of results we have clarified a few points in the revised manuscript. Our variability tests were conducted with n=3 while our comparisons of awake and isoflurane scans were done in males (n=3) and females (n=3). When no difference was observed between males and females we pooled the data so that all other analyses are based on n=6. We now state our n-values to remind the reader when results are backed by higher numbers. With this we hope the reviewer agrees that readers will find our interpretations to be backed by a sufficient amount of data.**

Line 368: please fix typo texttimes

**Done.**

Page 12, line 452, 460: Is RR in line 452 a general value? Or measured in your experiment?

Please add the measured RR values from awake state as comparison for the discussion about RRs	in anesthesia.

Line 452, The RR in line 452 is a general value refering to reference 35. A section about the awake RR has been added.

Page 13, line 473ff: the authors suggest awake mouse MT-31P-MRS should facilitate measuring the true, physiological ATP creation rates without isoflurane influence. This seems possible, but a few problems might be addressed as well, like limited scan time and MT-31P-MRS being notoriously long scans. Motion stability for longer scan times in ISIS might still become a problem, even if it's not actual head motion, but e.g. body motion during breathing or by fighting against the restraint, which could cause B0 fluctuations in the head. Please address these issues as well.

**This has shortly been addressed with an emphasis on feasibility.**

Line 479: Here authors claim, ATP suppression by anesthesia is substantial – however, that is not based on shown data nor is it backed by a reference. Please add support for that claim.

**We have elaborated this.**

Line 483 needs a closing paranthesis after the figure 7 reference.

**Done.**

Line 482ff: While the argument, that basic 31P MRS analysis was already able to show significant PCr decrease, a more fancy analysis might have been able to find more accurate ATP changes and PCr/ATP ratios as well. However, the small n=3 would still weaken those results, so maybe indeed leave it at this.

**This section has been removed, however the sample size was n = 6.**

Section “Methodological considerations”

In fMRI in rats and mice isoflurane does indeed vasodilate the brain vessels and leaves no no capacity to further open up the vessels for a increased blood flow in BOLD experiments, effectively canceling BOLD signal for isoflurane at 2%. However, a combination anesthesia of Medetomidine together with (very) low levels of isoflurane recover the BOLD responsiveness of the rodent brain. In comparison to your (low) isoflurane protocol, added Medetomidine might allow to lower isoflurane even further, maybe keeping the respiration rate >100, possibly also reducing the ATP suppression. Please add a discussion of regarding this well described anesthesia combination.

**Thank you for this. That is a very good point and we have added a brief section regarding this.**

Page 14: author contributions

Line 523: … surgeries, SM, SM and RWK performed ..... : the second SM is not required/wrong, please fix

**Done**

Support information:

The naming of the S1 File, S2 file is not the same as on the downloadable files, please fix

**Done.**

References:

Some references contain broken html formatting codes (e.g. 21 !sup?14 with ! And ? Inverted), please fix.

Also Ref 29, 71, 72, 88

**Done**

**Reviewer 2:**

The authors have performed 31P MRS in awake and isoflurane anesthetized mice. They use a mouse cradle suspension system they have previously developed and compare repeated measurements as well as awake versus anesthetize male and female mice. The sample sizes (number of animals) are rather low. However, for the purpose of this study it appears acceptable to present significant values based on such low sample numbers. The study is experimentally sound and has been performed according to the state-of-the-art. The authors state two aims of this study. First, they want to show that 31P MRS can be performed in awake mice. Second, they want to assess the effects of isoflurane anesthesia on 31P MRS by comparing awake versus anesthetized mice. Regarding the first aim, the study provides convincing evidence that 31P MRS in awake mice is feasible. Regarding the second aim, the relevance of the findings does not become fully clear and several aspects in the discussion lack the necessary depth.

Therefore, the following points should be addressed by the authors:

Major points:

1. The study compares awake mice with isoflurane anesthetized mice. Presentation of the results and its discussion creates the impression that these data allow for general statements about awake versus anesthetized investigations. However, only one defined anesthetic condition was used. While it may represent a widely used regimen, other studies may use different isoflurane concentrations, and more importantly other anesthetics. Especially medetomidine is becoming more and more popular. It should become clear from the paper that the investigations do not allow for general statements beyond the anesthetic regimen applied.

**We fully agree that our results are only representative for isoflurane and that other effect may be observed used other anesthetic agents. In the revised version we clarify this and avoid broad statements about anesthesia limiting our discussion to effects of isoflurane and benefits of awake MRI/S. This has also been made clear in the manuscript title.**

2. The discussion is very broad and lacks focus. It touches upon many aspects of physiological and metabolic differences between awake and anesthetized states and cites a large number of references. However, several aspects are only discussed superficially, not with the required depth. Some are only loosely linked to the presented investigations and do not support the conclusions. Of course, it is the authors choice what they want to discuss.

**We agree and have tightened the Discussion. We hope the reviewer agrees that the new version is concise and more focused.**

However, given the already large number of citations, I would recommend to focus the discussion on the relevant points, in order to avoid the paper (and refence list) to become exceedingly lengthy. Some examples:

a. One example is the discussion about brain clearance. This topic, if not omitted, requires much more in-depth discussion with a much wider scope of citations to address the topic adequately. (remark in this context: I do not see how ref. 44 fits on page 3).

**We agree and have limited the mention of these studies to summarizing sentences about MRI studies showing effect of isoflurane. We find the Miao study is relevant because it shows isoflurane to affect brain solute transport. This has been clarified in the text.**

b. Another example is cell swelling. If not omitted, it should be mentioned that altered diffusivity has been measured in dependence on brain activity (see DOI: 10.1371/journal.pbio.2001494 , 10.1126/science.1241224, 10.1186/s12987-023-00443-2 )

**We have removed the discussion about cell swelling for conciseness. However, we have added the last reference to the list of studies using MRI to show effects of isoflurane.**

c. Effects of other anesthetic drugs are not discussed in sufficient detail, but seem important for the conclusions.

**We agree but also acknowledge that this is a vast topic. For this reason we have kept the mention of these matters short and added references to add breadth to the discussion.**

d. PCr changes under isoflurane have previously been observed by 1H MRS (https://doi.org/10.1016/j.neuroimage.2012.12.020) which might be worth discussing.

**This has been added.**

3. The authors state that body temperature of the mice was kept constant. However, there are reports that brain temperature can differ substantially from the core temperature. This seems not unlikely in cases where the skull is exposed. Do the authors have any possibility to measure (or estimate) the actual brain temperature? Otherwise, no statements about brain temperature should be made and a potential variability should be discussed as a possible source of error.

**We agree and have added these considerations to the Discussion.**

4. The authors state that breathing rate varied between scans, but do not discuss the implications in sufficient detail. It has been shown previously that CO2 level depends on breathing rate, which in turn may have impact on pH. More discussion is needed how this may influence the obtained results.

**Regarding respiration rates, we report that (1) the anesthetized ISIS scan during the intra-variability test showed a significantly lower rate compared to the initial ISIS scan, (2) respiration rates in awake mice were within the range expected for non-stressed animals, and (3) the anesthetized respiration rate following the awake ISIS scan was stable, averaging 91 ± 5.1 breaths per minute.**

Minor points:

1. Which reference line was used for the 31P ppm scale?
**The ppm scale was set to PCr = 0 ppm which is in accordance with standard practice.**

2. Ref 52 requires updating

**Done.**
